# Pós-graduaáo na américa latina - um olhar para o futuro

**DOI:** 10.15649/cuidarte.3141

**Published:** 2023-05-27

**Authors:** Carolina Giordani da Silva, Marina Nolli Bittencourtt

**Affiliations:** 1 Universidade Federal do Mato Grosso, Brasil. Email: carolina.silva7@ufmt.br Universidade Federal do Mato Grosso Universidade Federal do Mato Grosso Brazil carolina.silva7@ufmt.br; 2 Universidade Federal do Mato Grosso, Brasil. Email: marina.bittencourt@ufmt.br Universidade Federal do Mato Grosso Universidade Federal do Mato Grosso Brazil marina.bittencourt@ufmt.br

A ciencia preocupa-se com a causalidade, buscando a compreensáo da realidade por meio da observado, verificado e experiencia. Neste sentido, os testes das hipóteses e a experimentado sáo considerados métodos científicos. Já a Filosofia preocupa-se com a finalidade da vida humana, a natureza do ser e da realidade, a teoria e os limites do conhecimento, tendo como a intuido, a introspecáo e o raciocinio exemplos de metodologias filosóficas. Ciencia e filosofia comungam do mesmo objetivo que é aumentar o conhecimento. Assim, a ciencia de qualquer disciplina está ligada a sua filosofía, que fornece a base para o entendimento e o desenvolvimento das teorias para a ciencia^1^.

A ciencia da Enfermagem é definida como o conhecimento substantivo, específico da disciplina, que enfoca o processo humano-universo-saúde articulado nas estruturas e teorias de Enfermagem. A filosofia da ciencia na Enfermagem ajuda a estabelecer o significado da ciencia por meio do entendimento e da avaliaáo dos conceitos, teorias, leis e metas de Enfermagem a medida que se relacionam com a prática assistencial. Destarte, o desenvolvimento do conhecimento de Enfermagem reflete a interface entre a ciencia e a pesquisa em Enfermagem, tendo por finalidade melhorar a prática de Enfermagem^1^^,^^2^.

Na trajetória de se afirmar enquanto ciencia, as pesquisas desenvolvidas na Enfermagem, no cometo do seu desenvolvimento científico foram, predominantemente, estudos quantitativos, muito influenciados pelo paradigma positivista, no empirismo lógico, acompanhando uma tendencia já consolidada pela Medicina. Entretanto, estes estudos, por si só, nao refletiam os aspectos humanos, subjetivos e singulares, que permeiam os diferentes contextos de cuidado, deixando, ainda, lacunas a serem respondidas.

Ressalta-se, neste sentido, a importancia dos programas de Pós-Graduaáo disseminados nas diferentes universidades no mundo, contribuindo para o desenvolvimento do conhecimento da ciencia e filosofia da Enfermagem, que precisam dar respostas as demandas sociais em direáo a uma atenáo de qualidade e resolutividade.

Nessa perspectiva, e frente ao contexto das discussóes atuais, além da importancia de fortalecer a nossa ciencia nas pesquisas de Enfermagem por meio da proposito de soluóes inovadoras e empreendedoras para o cuidado, nos deparamos, especialmente nos países da América Latina, com as questoes sociais que urgem o fortalecimento de pesquisas que deem atenáo para os Objetivos do Desenvolvimento Sustentável (ODS) proposto pela Organizado das Naóes Unidas para a promodo da saúde e da saúde mental, em uma regiáo com ainda muitas vulnerabilidades sociais.

Considerando a (o) enfermeira (o) como líder da promodo da saúde, a pós-graduaáo tem um papel importante na proposido de pesquisas com impacto social, a luz dos ODS, que proponham intervendes que promovam a saúde dessa populado atino-americana que, apesar das grandes desigualdades, está envolta de grandes riquezas naturais e culturais que podem ser aliadas nessa proposido, e que permitiráo que os programas de pós-graduado em Enfermagem nesses países atendam ao chamado da saúde planetária.

Assim, pensando que a ciencia tem que responder a uma determinada realidade, que na américa latina é permeada pelas desigualdades, pela pobreza, corrupáo, desmatamento, e políticas públicas ineficazes, mas também pelas riquezas naturais e culturais, para os pesquisadores de Enfermagem o desafio na produdo de conhecimento e novas tecnologias que atendam aos ODS e que promovam a saúde local e planetária é grande, entendendo que o meio ambiente está intrinsecamente conectado e interligado a promodo e manutenáo da saúde.

Entende-se, nesse sentido, que é essencial que os PPG tenham também uma atuaáo política frente a realidade atual, de forma que nossas pesquisas possam apresentar a sociedade e aos governantes dados baseados em evidencias em diredo a caminhos que podemos seguir para termos sucesso no desenvolvimento sustentável de nossa populado.
